# Iron Deficiency Caused by Intestinal Iron Loss—Novel Candidate Genes for Severe Anemia

**DOI:** 10.3390/genes12121869

**Published:** 2021-11-24

**Authors:** Carolina Huettmann, Matthias Stelljes, Sugirthan Sivalingam, Manfred Fobker, Alexis Vrachimis, Anne Exler, Christian Wenning, Carola Wempe, Matthias Penke, Andreas Buness, Kerstin U. Ludwig, Martina U. Muckenthaler, Andrea U. Steinbicker

**Affiliations:** 1 Department of Anesthesiology, Intensive Care and Pain Medicine, University Hospital Muenster, University of Muenster, 48149 Muenster, Germany; carolina.huettmann@uni-muenster.de (C.H.); carola.wempe@ukmuenster.de (C.W.); 2 Medical Clinic D: Hematology, Haemostaseology, Oncology and Pneumology, University Hospital Muenster, University of Muenster, 48149 Muenster, Germany; Matthias.Stelljes@ukmuenster.de; 3 Core Unit Bioinformatics Data Analysis, Medical Faculty, University of Bonn, Venusberg-Campus 1, 53127 Bonn, Germany; s.sivalingam@uni-bonn.de (S.S.); buness@uni-bonn.de (A.B.); 4 Institute for Medical Biometry, Informatics and Epidemiology, Medical Faculty, University of Bonn, Venusberg-Campus 1, 53127 Bonn, Germany; 5 Institute for Genomic Statistics and Bioinformatics, Medical Faculty, University of Bonn, Venusberg-Campus 1, 53127 Bonn, Germany; 6 Center for Laboratory Medicine, University Hospital Muenster, University of Muenster, 48149 Muenster, Germany; Manfred.Fobker@ukmuenster.de; 7 Department of Nuclear Medicine, University Hospital Muenster, University of Muenster, 48149 Muenster, Germany; Alexis.Vrachimis@goc.com.cy (A.V.); anne.exler@ukmuenster.de (A.E.); christian.wenning@hospital-lingen.de (C.W.); 8 Oncological Clinic, St. Franziskus Hospital, Franziskusstraße 6, 49393 Oldenburg, Germany; p.onkolohne@gmail.com; 9 Institute of Human Genetics, University Hospital Bonn, Medical Faculty University of Bonn, 53113 Bonn, Germany; kerstin.ludwig@uni-bonn.de; 10 Department of Pediatric Oncology, Hematology and Immunology, University of Heidelberg, 69117 Heidelberg, Germany; Martina.Muckenthaler@med.uni-heidelberg.de; 11 Molecular Medicine Partnership Unit (MMPU), 69117 Heidelberg, Germany; 12 Department of Anesthesiology, Intensive Care Medicine and Pain Therapy, University Hospital Frankfurt, Goethe-University Frankfurt, 60590 Frankfurt am Main, Germany

**Keywords:** red cells, iron, erythropoiesis, genetically caused iron imbalance, intestinal iron loss

## Abstract

The adult human body contains about 4 g of iron. About 1–2 mg of iron is absorbed every day, and in healthy individuals, the same amount is excreted. We describe a patient who presents with severe iron deficiency anemia with hemoglobin levels below 6 g/dL and ferritin levels below 30 ng/mL. Although red blood cell concentrates and intravenous iron have been substituted every month for years, body iron stores remain depleted. Diagnostics have included several esophago-gastro-duodenoscopies, colonoscopies, MRI of the liver, repetitive bone marrow biopsies, psychological analysis, application of radioactive iron to determine intact erythropoiesis, and measurement of iron excretion in urine and feces. Typically, gastrointestinal bleeding is a major cause of iron loss. Surprisingly, intestinal iron excretion in stool in the patient was repetitively increased, without gastrointestinal bleeding. Furthermore, whole exome sequencing was performed in the patient and additional family members to identify potential causative genetic variants that may cause intestinal iron loss. Under different inheritance models, several rare mutations were identified, two of which (in *CISD1* and *KRI1*) are likely to be functionally relevant. Intestinal iron loss in the current form has not yet been described and is, with high probability, the cause of the severe iron deficiency anemia in this patient.

## 1. Introduction

Anemia is a common disorder and represents a major burden of disease worldwide. According to Kassebaum et al., more than 2 billion people are affected by it, including asymptomatic or mildly affected patients. While anemia might occur in people of all ages, a higher incidence has been reported in children younger than 5 years of age, pregnant women, and elderly people (>70 years) [1]. The most frequent types of anemia are (i) iron-deficiency anemia (IDA), affecting about 50% of anemic people, and (ii) anemia of chronic disease (ACD, >40%). ACD is characterized by elevated inflammatory parameters but normal to elevated ferritin levels [2]. In contrast, anemias caused by genetic defects encompass a heterogeneous group of rare disorders. Recently, Gulbis et al. estimated the prevalence of rare anemias defined as less than one case per 2000 [3]. More than 80% of those rare anemias follow an autosomal-dominant inheritance pattern, which includes a probability of 50% for children to inherit the disease-causing allele. Up to date, there are more than 90 rare anemias listed in the “European Network of Rare Congenital Anemias” [4].

Rare anemias are challenging for both patients and the physicians involved in their treatment. The underlying molecular pathology often remains largely unknown. In the last few years, new avenues of research have opened up with the advent of novel technologies, including high-throughput sequencing and characterization of mutations using animal models. Mutations in iron-related genes are rare, may cause anemia, and have been reviewed in [2]. Hepcidin is the iron regulatory hormone that inhibits intestinal iron absorption and the release of iron into the circulation from iron storage cells, such as macrophages and hepatocytes, by internalization and degradation of the iron absorption channel ferroportin. Hepcidin production is transcriptionally regulated by inflammatory cytokines, including interleukin-6 (IL-6), and bone morphogenetic protein (BMP) signaling. By the induction of cytokines, anemia of inflammation, also named anemia of chronic disease, develops. Other regulators include iron, hypoxia, transforming growth factor β (TGFβ), tumor necrosis factor α (TNFα), hemojuvelin (HJV, a BMP co-receptor), activin B, and the unfolded protein response [5,6,7,8].

Here we present a patient with severe iron deficiency anemia. Routine diagnostics were not conclusive in regard to the cause of the anemia. Extensive further diagnostics led to the hypothesis of a genetic disorder. The results of the genetic analysis are discussed in this paper and stress the importance of further research of iron metabolism disorders in order to improve diagnostics and treatment options.

## 2. Materials and Methods

Informed consent for blood analyses, genetic analysis, and to report the patient’s case anonymously has been given by the patient himself and all family members involved in the study. The patient’s clinical history was taken through (i) personal and family interviews, (ii) assessment of patient records at the University Hospital Münster, and (iii) additional information in records of the patient’s general practitioner and hematologist/oncologist, all of which was accessed as approved by the patient and his family members.

### 2.1. Measurement of Red Blood Cell Production by Intravenous Injection of Radioactively Marked Autologous Red Blood Cells

Quantitative iron kinetic studies are used to assess different parameters of hematopoiesis. About 350 kBq (9.5 µCi) of ^59^Fe citrate was incubated in the patients’ plasma (13 mL) and re-injected intravenously. Serial whole blood and plasma samples were obtained for 10 days, starting from the time of intravenous injection, to determine ^59^Fe incorporation into the erythrocytes (*n* = 6 in the first 90 min post injection, *n* = 10 for 10 days in 24-h intervals post injection). The samples were measured in a well scintillation counter and normalized to 1 mL. Radioactivity was expressed in counts per minute (cpm). Plasma iron clearance and plasma iron utilization were calculated as described previously [9,10,11]. At the same time, body surface counting of the following organs/body regions was performed for 10 days: liver, spleen, and sacrum (as a reference region for the bone marrow) using a gamma scintillation probe in a room with low background radioactivity expressed in cpm. In addition, hematocrit was determined from every blood sample. In case of stability, blood loss can be excluded, in case of a decrease, blood loss or an increase in fluids are potential causes.

### 2.2. Measurement of Iron Excretion in the Urine and Intestinal Iron Excretion

In another set of experiments, samples from feces were taken for seven days for the detection of a possible gastrointestinal iron loss, measured in a well scintillation counter, normalized to 1 mL volume, and expressed in cpm.

In detail, stool samples (~0.1 g) were dried at 70 °C for 3 days until a constant mass was measured. An amount of 5 mg of this dry mass was mixed with 100 µL 65% (*w*/*w*) nitric acid (“Ultrapure”, Merck, Darmstadt, Germany) and digested at 25 °C overnight. After an incubation for 90 min at 70 °C, the lysate was cooled and centrifuged. An amount of 5 µL of the sample was diluted with 995 µL ultrapure water.

Measurements were performed using an AA-6300–Shimadzu atomic absorption spectrophotometer (Shimadzu Corporation, Kyoto, Japan) with graphite furnace atomization, equipped with a deuterium lamp for background correction and an iron hollow-cathode lamp (Typ L233-26NB (Hamamatsu Photonics, Herrsching, Germany), operated at a current of 12 mA and a wavelength of 248.3 nm, with a spectral band pass of 0.2 nm. Samples of 10 μL were injected into high-density graphite tubes (Shimadzu) using a Shimadzu ASC-6100F autosampler. Calibration curves were prepared using five concentrations (0–20 µg/L Fe), with the linear correlation coefficients obtained ranging between 0.99–0.9943. The graphite furnace program for iron determination was temperature (°C)/time (s) for drying, step 1 (200)/20, drying step 2 (500)/10, ashing (1200)/5 in ramp mode and atomization (2500)/5, and cleaning (2700)/2 in the step mode, respectively, with an argon flow rate of 250 mL min^−1^. The accuracy of the quantitative iron determination was assured by simultaneous analysis of certified reference material (“Liquid Assayed Multical”, Bio-Rad Laboratories GmbH, München). 

For stool hemoglobin measurement, 0.5 g feces in 1 mL 0.9% NaCl solution was homogenized with a T-25 ULTRA-TURRAX (IKA-Werke, Staufen, Germany) for 2 min and centrifuged for 5 min (at 1000 g). For hemoglobin determination, the supernatant was measured by a cyanohemoglobin method described by Tapernon et al., 2001 [12] on a Roche Cobas c502 clinic chemistry analyzer (Roche Diagnostics GmbH, Mannheim, Germany).

### 2.3. Statistical Methods

All values were expressed as mean ± S.D. Data were analyzed using students *t*-Test and one-way ANOVA Kruskal-Wallis test with Dunn’s correction for multiple comparisons, when applicable. Statistical significance was considered for *p* values < 0.05.

### 2.4. Genetic Analysis of Patient and Relatives

Whole exome sequencing libraries were prepared with SureSelectXT Target Enrichment System for the Illumina Platform V4 (Agilent, Santa Clara, CA, USA), according to manufacturer’s protocols. They were sequenced in paired-end 75 bp mode on an Illumina NextSeq 500 deep sequencing instrument. Library enrichment was performed in the University Hospital Heidelberg. After sequencing, fastq-data was transferred to the Core Unit Bioinformatic Analysis (CUBA) at the University Hospital Bonn, where alignment, variant calling, and annotation was performed using GATK version 4.1.0 and Annovar version v2019-10-24.

In the first step, variants were only retained in the analysis if a minimal depth of 20X at that position was observed. Subsequently, the variant list was filtered according to several strategies to identify the likely causal variants in the family. Given the pedigree structure, different inheritance modes were considered. As mentioned above, both mother and sister have been or are still affected by anemia. Phenotypically, they differ from the patient. However, the possibility remains that they too carry the same mutation. On the other hand, the patient’s father and brother are not anemic, meaning their analyses were used to exclude a certain number of variants.

**Figure 1:**
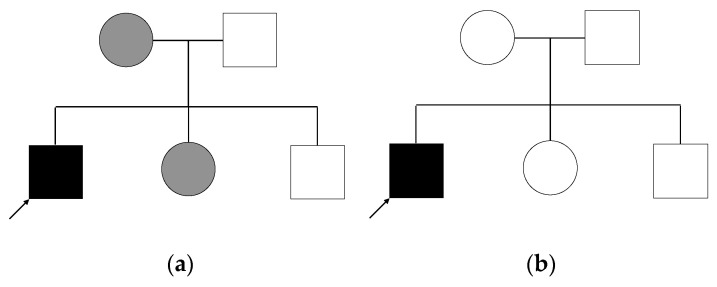
Pedigree. Two different family trees are plausible, looking at the different modes of inheritance. (**a**) A potential family tree considering that mother and sister are affected by the same type of anemia as the patient. The index patient (**arrow**) presents a unique clinical phenotype (**black square**), while mother and sister (**grey circles**) present with a different, less severe anemia. In this scenario, identical causal variants are expected in the three affected persons. (**b**) The possible family tree considering that only the index patient (**arrow**) is affected by the unknown anemia, whereas his mother and sister suffer from a different type/types of anemia. The index patient is the sole carrier of the genetic mutation.

Considering the possibility that the patient, his mother, and his sister carry the same variation (Figure 1a), the following inheritance modes are plausible:

1. Autosomal dominant inheritance with variable expression of the phenotype. In this case, each affected individual carries the variant heterozygously. We do not consider variants for which the mother is homozygous, as this is unlikely given the expected rarity of the variant.
2. X-linked inheritance: the patient is a hemizygous carrier of the variant, while mother and sister are heterozygous.
3. Autosomal dominant inheritance with a second hit in the patient: as mentioned above, patient, mother, and sister all carry the same variant, but in addition to that, the patient carries a second variant that the others do not have, and which would explain the increased severity of the patient.

In all three possible cases, neither the father nor the brother carried the variant.

Under the assumption that the mother’s and sister’s phenotypes are etiologically independent from the patient’s (i.e., phenocopies, Figure 1b), the following inheritance modes are possible:

1. De novo mutation: the patient carries a variant in a heterozygous state, that he shares with no other individual in the family.
2. Autosomal recessive inheritance: the patient is a homozygous carrier of the variant, while both parents are heterozygous. His siblings either do not carry the variant or are heterozygous.
3. Compound heterozygosity: in case of compound heterozygosity, the patient carries two different heterozygous variants in the same gene, each inherited by one parent. The not affected father as well as both siblings could each be carriers of one of the variants. The mother and sister could also each be carriers of one variant.

In the third step, common variants were excluded based on variant information from non-Finnish Europeans, as provided by gnomAD [13]. Only those variants with a frequency between 0 and 0.0001 were included. Finally, the CADD13 filter was applied to assess the likelihood of a variant being pathogenic. Only those variants with a Phred-score of 20 or higher were considered relevant.

## 3. Results

### 3.1. Patient History

Born in 1970, male. The anemia has been known since 1996. Comorbidities are arterial hypertension, obesity, and hyperlipoproteinemia. He is neither vegan nor vegetarian, but he takes regular European food including meat. 

In July 2010, the patient suffered from a myocardial infarction. He received percutaneous transluminal coronary angioplasty (PTCA) and one drug eluting stent. After the intervention, intermittent atrial fibrillation occurred. In October 2010, he collapsed at his workplace due to low hemoglobin levels, was hospitalized, and received about 40–50 red blood cell concentrates (RBCs). Anamnestically, Hb values were about 5 g/dL. After his release from the hospital, he received blood transfusions on a weekly basis until the end of 2011. He still presented with iron deficiency and low ferritin levels.

In January 2012, a second myocardial infarction occurred and was treated again with PTCA and a placement of a drug eluting stent. By that time, the anemia treatment consisted of blood transfusions every two weeks to maintain Hb levels between 8 and 9 g/dL. The treatment was switched to intravenous iron supplementation (iron(III)hydroxide-sucrose-complex) every 3–4 weeks. Since 2013, the supplementation consisted of 1000 mg iron carboxymaltose every 3 weeks. Under this therapeutic regimen, two episodes of low Hb occurred, which presented with general weakness, paleness, tachycardia, and tinnitus. The Hb was measured between 5 and 6 g/dL.

In December 2013, the patient was seen by his general practitioner with low Hb of 6.6 g/dL, HCT of 20%, and reported black feces and was sent to the hospital. He received five red blood cell concentrates, 1000 mg iron carboxymaltose, and underwent a gastroscopy and colonoscopy. No causes of intestinal bleeding were found.

Subsequently, the University Hospital Münster was approached for further investigation. At that time, the Hb was 10.9 g/dL, and ferritin levels were at 39 µg/L. The medication consisted of candesartan/hydrochlorothiazide 8 mg/12.5 mg 1-0-0, ASS 100 mg 1-0-0, simvastatin 40 mg 0-0-1, molsidomin 8 mg 1-0-0.5, and pantoprazol 40 mg 1-0-1.

During the following years, the patient received 1–2 red blood cell concentrates and 500–1000 mg iron carboxymaltose per month (e.g., January 2014: 500 mg iron carboxymaltose; February 2014: 1000 mg iron carboxymaltose and 1 RBC at a hemoglobin of 9.9 g/dL; March 2014: 2 RBCs and 1000 mg iron carboxymaltose resulted in a hemoglobin of 10.6 g/dL). The results of laboratory testing over years, to observe the patient’s clinical state and to exclude differential diagnoses, are summarized in Table 1 and Table 2. In addition, the laboratory results of the last three years are shown in Table 3. Between 2019 and 2021, the patient received 500 mg iron carboxymaltose about every six weeks intravenously but did not require RBCs. In addition to laboratory testing, multiple bone marrow biopsies and skin biopsies were performed. These results are outlined in Table 4. In addition, the presented patient has had multiple colonoscopies, esophago-gastro-duodenoscopies, and a gastrointestinal scintigraphy. All investigations excluded gastro-intestinal bleeding.

### 3.2. Family History

*Mother:* Born in 1950. She was first diagnosed with anemia in the 1990s. The type of anemia was not further specified. She was treated with oral iron supplements, and over the course of therapy, the anemia recovered. No blood transfusions were ever necessary. Current medication consists of metformin, insulin, and an unknown medication for arterial hypertension. The Hb on 23/10/12 was 12.7 g/dL.

*Father:* Born in 1942. No anemia reported.

*Sister:* Born in 1974. She was diagnosed with chronic polyarthritis and sarcoidosis in 1999. Reported anemia of unknown specification with Hb as low as 5 g/dL. The treating rheumatologist tolerates the anemia. No blood transfusions so far. The current medication consists of corticosteroids, azathioprin, diclophenac, and golimumab. She has two children, one daughter and one son.

*Brother:* Born in 1980. No anemia reported.

### 3.3. Measurement of Red Blood Cell Production by Intravenous Injection of Radioactively Marked Autologous Red Blood Cells

**Figure 2:**
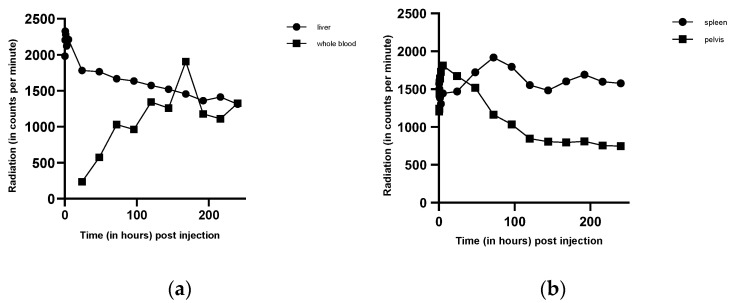
Radiation measured over selected organ systems. After injection of radioactively labeled ^59^Fe, radiation was measured in the liver and whole blood (**a**). After an initial increase of radiation in the liver, a continuous decrease was observed. Radiation in whole blood, however, increased over time, indicating intact hematopoiesis, followed by the release of ^59^Fe loaded erythrocytes into the bloodstream. (**b**) The measurement of radiation over spleen and pelvis (representing the hematopoietic system) is seen in Figure 2b. While the splenic radiation remains stable over time, radiation in the pelvis decreases, an indicator of hematopoiesis and release of ^59^Fe into the blood (see Figure 2a).

**Figure 3:**
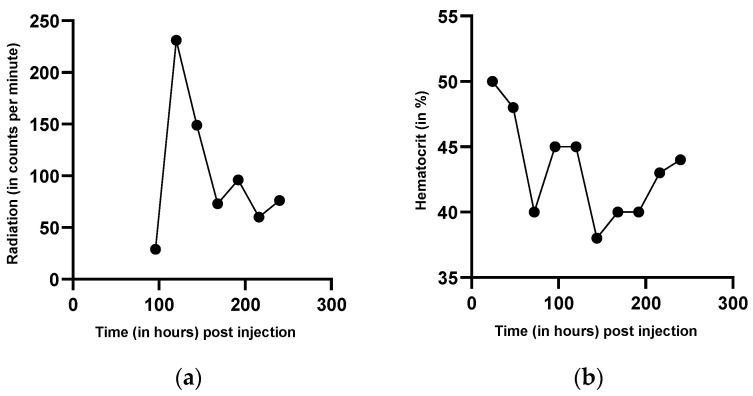
Measurement of (**a**) radioactivity in feces and (**b**) hematocrit (HKT) during the ferrokinetic study. Figure 3a shows the radiation measured in feces after the administration of ^59^Fe. After the first measurement, radiation rose and afterwards decreased continuously. Hematocrit (Figure 3b) was measured during the ferrokinetic study to exclude a hemorrhage as a possible source of intestinal iron loss. The hematocrit levels remained stable during the ferrokinetic study.

Plasma iron clearance was calculated with 44 min, which is below the normal time of 70–140 min. Blood volume was estimated with 5.8 L blood. The measurement of iron utilization in the liver and whole blood (Figure 2a), and the spleen and bone marrow (pelvis) (Figure 2b), indicated regular iron utilization with a maximum on day 7. There was an early increase of the radioactive signal in bone marrow and in the liver, followed by a continuous decrease. Activity in the spleen, in contrast, increased on day 2, decreased afterwards, and showed undulations during the following days. There was a peak in whole blood measurements on day 13, which remains without explanation. Radioactivity was also determined in stool samples. Here, signal intensity suddenly increased with a peak on day 11 (Figure 3a).

During the measurement, hemoglobin and hematocrit were determined every day. Although serum iron levels decreased, hematocrit (Figure 3b) and hemoglobin remained stable, indicating that the loss of iron was not due to bleeding.

### 3.4. Iron Levels in Urine

Iron levels in urine were determined as follows: Iron in urine: 4.7 µg/L. Creatinine in urine: 80.92 mg/dL = 4.7 µg/10dL × 1dL/80.92 mg = 4.7/809.2 = 0.00580 µg/mg creatinine (normal range: 30–240 µg/mg).

### 3.5. Iron Levels in Feces

**Figure 4:**
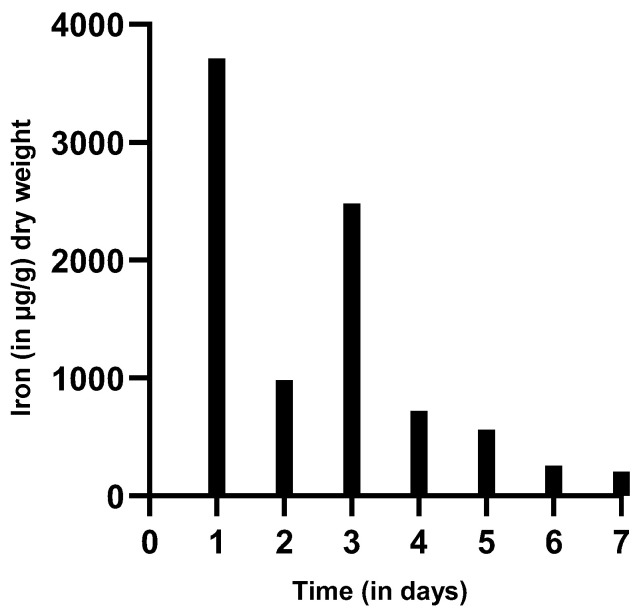
Measurement of iron content in feces (in µg/g dry weight). Iron content of feces was determined after application of ^59^Fe on day 0 and measured daily for 7 consecutive days. The iron content was highest on day one, and with one exception on day 2, it continuously decreased.

**Figure 5:**
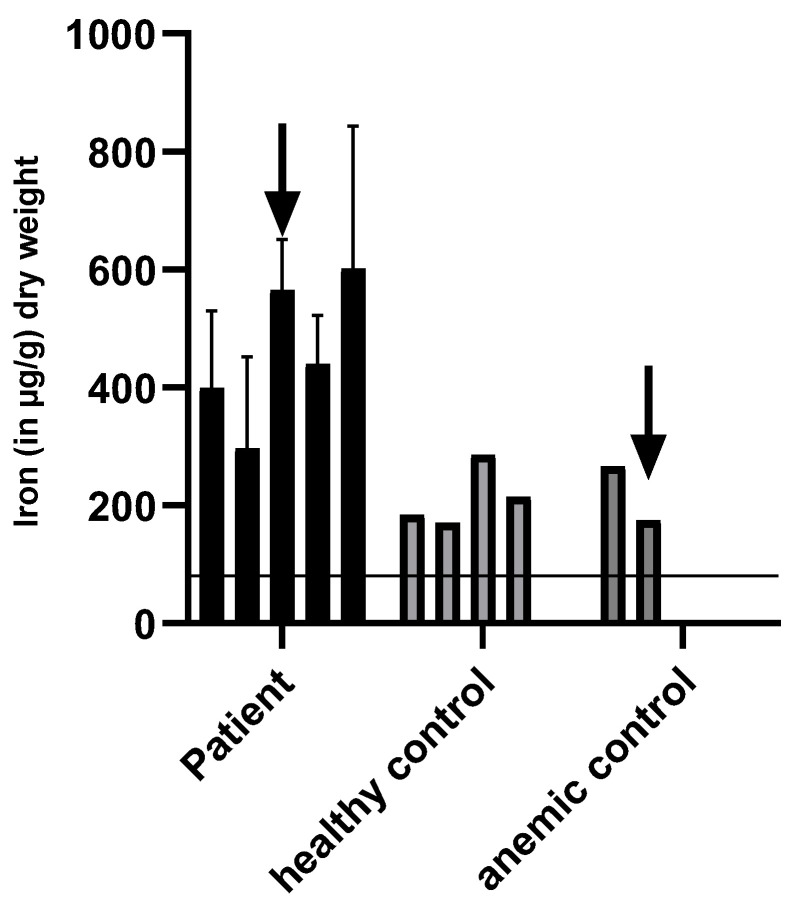
Measurement of iron in feces (in µg/g dry weight). Iron excretion in feces was measured in the patient (**left**) in irregular intervals for a total of 32 days. The arrow indicates measurements after the administration of 1000 mg i.v. iron carboxymaltose on day 16. These results were compared to the iron excretion of a healthy individual (**middle column**) with no iron administration and an anemic (**right column**) individual, who received 1000 mg of carboxymaltose iron. The arrow indicates measurements after the intravenous iron administration. The horizontal line indicates the average iron loss via feces in humans, which is about 100 µg iron/g dry weight feces.

Iron levels in µg iron/g dry weight of feces were determined via direct measurement in feces. After radioactive ^59^Fe application, the patient excreted 3712 µg iron/g dry weight of stool. On day 2, 980 µg iron/g dry weight was detected, followed by another increase on day 3, with 2480 µg/g dry weight. From day 4–7, iron excretion in feces decreased from 720 to 560 to 255 and 205 µg iron/g dry weight of stool (Figure 4). No hemoglobin (detection level <20 mg/L) was determined in the patient’s sample. As radioactive iron might be excreted differently, feces samples of the patient were analyzed prior to and after 1000 mg intravenous carboxymaltose supplementation. Prior to and after intravenous iron supplementation, iron excretion in the patient’s feces was higher than in healthy adults without iron supplementation or anemic patients with iron supplementation (Figure 5).

### 3.6. Results of Whole Exome Sequencing

Initially, using multisample variant calling, 26,040 genetic variants were found in at least one member of the family. After filtering, 347 possible variants remained, which were at least heterozygous in the index patient, consisted of 20 reads or more, and were rare in the patient’s population. Afterwards, the different inheritance modes were applied to these results. Of the six inheritance modes, three (X-linked inheritance, compound heterozygosity, and autosomal dominant inheritance with second hit in the patient) were excluded, as no variant matched the respective criteria. The variants potentially causative, according to the four distinct genetic models, are provided in Table 5 and Table 6. In the discussion, the candidate genes are analyzed, distinguished, and discussed. Finally, the two most probable causes are identified.

Following the results of the genetic analysis, literature research was performed to determine the function of the respective gene and thus the relevance in regard to anemia and iron metabolism, as seen in Table 7.

## 4. Discussion

In the current manuscript, we present in detail a patient’s case with increased intestinal iron excretion. Sequencing of the patient and additional family members revealed 18 genes with rare mutations that represent candidate genes for causing the intestinal iron excretion, two of which could be prioritized based on literature review.

### 4.1. Discussion of Anemia Causes

The majority of patients who present with decreased hemoglobin levels suffer from iron deficiency. The patient presented with a prolonged iron deficiency that could not, even with high amounts of iron, be replenished. The second most common form of anemia is ACD. Iron absorption and iron utilization is inhibited due to inflammation or cancer. In the presented case, infection was not an issue. If iron was supplemented in high doses, hemoglobin could be maintained at normal to low levels. In ACD, the hepatic hormone hepcidin is induced and causes degradation of the iron exporter ferroportin [32,33]. Hepcidin levels of the patient were very low. Suppression of hepcidin also indicates that iron deficiency is the leading cause. Only about 6–8% of anemias are so-called rare anemias. These include hemolytic anemias, such as hemoglobinopathies that are caused by inherited defects either in the structure of Hb, in Hb synthesis, or spherocytosis. Alterations of the shape and/or viability of erythrocytes cause acute or chronic hemolysis [3]. The presented case did not present with hemolysis. Hemoglobin synthesis was intact. Of the patients with rare anemias, less than 1/1000 present with a genetic mutation. The identification of novel genes involved in iron transport and homeostasis include mutations in genes that control (1) duodenal iron absorption (e.g., *DMT1* [34]), (2) systemic iron homeostasis (e.g., *TMPRSS6*), or (3) erythroid iron absorption and utilization. These mutations were not present in the current case.

Erythroid precursor cells satisfy their high iron demand by receptor-mediated endocytosis of Tf(transferrin)-bound iron via TfR1 (transferrin receptor1). Therefore, mutations in genes involved in endosomal iron uptake or export, such as *DMT-1* [35], the endosomal ferrireductase *STEAP3* [36], or *Sec15L1* [37,38] cause erythroblast iron deficiency and anemia. In the cytoplasm of the erythroblast, iron is stored in form of ferritin or utilized for heme or FeS cluster biogenesis in mitochondria. Thus, mutations in genes that interfere with mitochondrial iron transport (e.g., mitoferrin [38]) or FeS cluster biogenesis (e.g., *GLRX5* [39,40], *ABC7* [41,42]) can cause microcytic anemias in animal models and/or patients. For example, mutations in *ALAS2*, the first enzyme of the heme biosynthesis pathway, cause x-linked sideroblastic anemia due to a reduction in the synthesis of PPIX (protoporphyrin IX). Consequently, iron accumulates in erythroblasts, which triggers ROS production and cellular damage. Similarly, loss of the mitochondrial glutaredoxin *GLRX5* causes mitochondrial iron accumulation, oxidative stress, and impaired erythrocyte function [39,40]. To date, a single patient with *GLRX5* inactivating mutation has been described. The *GLRX5* mutated patient is hallmarked by mild microcytic anemia, iron overload, and ring sideroblasts. The Hb value dropped in the course of his life until he was transfusion dependent. Interestingly, iron chelator treatment could resolve his anemia [43]. All these genes do not contain relevant variants.

A genetic mutation also causes iron-resistant iron deficient anemia (IRIDA). Genetic IRIDA is a microcytic anemia resistant to both oral and parenteral iron supplementation [44]. It is caused by mutations in the *TMPRSS6* gene encoding matriptase-2. Patients with IRIDA, and mice with impaired function of matriptase-2, show inappropriately high hepcidin levels [44,45]. *TMPRSS6* is a serine protease that cleaves HJV, and thereby inhibits hepcidin induction [46]. The patient presented without a mutation in *TMPRSS6*, as well as with low hepcidin values.

In conclusion, the presented case was a case of rare anemia with unknown origin.

### 4.2. Discussion of Clinical Observations from a Hematological Point of View

Examination of the peripheral blood and the bone marrow was done repeatedly, including microscopic evaluation of blood and bone marrow smears, bone marrow histology, and screening for genetic aberrations, known to be associated with hematological diseases. With only unspecific findings, no significant signs of dysplasia in the bone marrow cytology, a normal karyotype, and no mutations detected for the genes ASXL1, CBL, DMT3A, EZH2, JAK2, RUNX1, SF3B1, SRSF2, TET2, TP53, U2F1 or ZRSR2, a hematological disease, e.g., a myelodysplastic syndrome, could not be diagnosed. Given the repeatedly analyzed hematological examinations performed between 2010 and 2015, adding no further diagnostic results, a systemic hematological disease could not be excluded. However, with stable clinical conditions and no new diagnostic findings until 2020, a systemic hematological disease remains unlikely in this case.

### 4.3. Discussion of Radioactive Measurement

The plasma iron clearance was clearly reduced. Increased iron uptake in bone marrow and liver indicates that hematopoiesis occurred appropriately in these organs. The following decrease in the time activity curves is a normal reaction of hematopoiesis, mainly due to “erythrocyte release”, which indicates a high production of erythrocytes in the bone marrow and their release into the blood stream. The increasing splenic activity, in contrast, does not indicate erythropoiesis; it is rather a sign for erythropoietic degradation in the spleen [47]. The peak of radioactivity in whole blood at day 13 remains without explanation and might be due to a single mismeasurement. The suddenly increased activity in the stool sample on day 11 (Figure 4) demonstrates a gastrointestinal iron loss, which apparently occurred suddenly and not continuously. Although serum iron levels decreased, hematocrit (Figure 4) and hemoglobin remained stable, signs for isolated iron loss without bleeding.

To conclude, the patient did not lose iron by increased plasmatic iron clearance and had intact hematopoiesis. Although hematocrit and hemoglobin remained stable, the iron loss in stool was increased.

### 4.4. Discussion of Intestinal and Urinary Iron Excretion

The endogenous excretion of iron by stool and urine was often examined in the context of food ingestion or intravenous injection of radioactive iron [48]. The biological half-life of iron in the body calculated from this investigation was 4–7 days [49]. Iron homeostasis is controlled mainly by iron absorption. It is generally accepted that endogenous excretion via feces and urine plays with 1mg/d loss of iron a minor role for the loss of the metal [50,51,52]. So far, ferroportin is the only known mammalian cellular exporter of iron. However, ferroportin is not expressed in the apical membrane of intestinal cells, it is only found in the basolateral membrane. No mechanism for controlled iron excretion has been described yet. Some manuscripts contradict the hypothesis of ferroportin expression at the apical membrane and refer to the intestine as an active part in the elimination of body iron [53,54,55]. High levels of iron in the feces are predominantly described in conditions with low absorption of iron, such as a loss-of-function mutation of DMT1, which is normally involved in iron absorption in the apical membrane in the intestine [34]. Other conditions with impaired intestinal iron absorption include a deletion of intestinal ferroportin, leading to severe anemia and iron accumulation within the enterocytes. Furthermore, a decreased activity of hephaestin, a multicopper ferroxidase, causes anemia and impaired dietary iron absorption [56]. In the genes, encoding these particular structures, no mutations were detected in the current, investigated patient.

The result of urinary iron in our patient indicates that iron excretion is strongly reduced. The low level of iron in the urine of the patient is in line with findings, that urinary iron loss depends on the concentration of iron in blood [57]. Increased urinary iron excretion is found in patients with systemic iron overload such as HFE-HH (HFE associated hereditary hemochromatosis) and β-thalassemia major, as well as in patients with tubular dysfunction [58].

Further investigation on iron cellular excretion mechanisms is necessary, as this study strongly suggests a so far unknown form of iron loss.

### 4.5. Discussion of WES

As prior diagnostic assessment of known causative genes such as *TMPRSS6* did not reveal any mutation, the patient’s DNA was systematically analyzed using WES. After stringent filtering, we identified 16 genes with rare mutations, which we discuss here. Of note, the results are limited by the restrictions that were performed by adjusting the called variants. Stringent filters were applied in order to ensure quality of the variants and to exclude likely benign and common variants. However, it remains possible that relevant variants have been excluded by the filtering process. Furthermore, we here focused on small variants/single base pair mutations and did not consider larger structural aberrations.

To prioritize genes among the candidates, we analyzed if the gene had been described previously in the context of iron pathways. Therefore, literature research was performed at first through the European Network for Rare and Congenital Anemias [4], to determine genes, that are known to have an impact on iron metabolism and anemia. They were included in the analysis and are listed as follows: *SLC40A1, FTH1, FTL, TFRC, TFRC2, SCARA5, HEPH, CYBRD1, HFE, ALAS2, ABCB7, GLRX5, SLCA38, STEAP3, CP, TF, SLC11A2, CDAN1, SEC23B, KIF23, KLF1,* and *GATA1*. However, none of those truly fitted the clinical presentation of the patient, and no potential causal variant was identified in any of these genes in our patient.

In the literature review of all candidates listed in Table 7, two of those turned out to have the strongest possible clinical relevance in regard to the presented case: *CISD2* and *KRI1*.

*CISD2* is a gene located on chromosome 4q and encodes NAF-1, a [2Fe-2S] cluster protein which is found in the mitochondria associated membrane, which connects the endoplasmatic reticulum and the outer mitochondrial membrane [20]. Of course, the other candidate genes might also be causative, especially the Zinc finger CCCH domain-containing protein 3 (ZC3H3).

Its main function is the inhibition of autophagy in cells and the promotion of longevity. *CISD2*, also known as *ERIS* or *Miner1*, belongs to the recently discovered family of *CISD* genes, which contain a unique CDGSH amino acid sequence in their Fe-S cluster binding domain called the NEET-fold. The human genome consists of three *CISD* genes (*CISD1-3*). *CISD2* has a homodimeric structure, in which each protomer contains one [2Fe-2S] cluster bound to the protein by a 3-Cys-1-His coordination geometry, shows a similar fold as its paralog mito-NEET (*CISD1*). At the chromosome level of the *CISD* gene, the mutation is found in a region associated with neural development [20]. NEET proteins are described to play an ancient role in cells associated with iron metabolism [20].

Cell models showed that NAF-1 plays an important role in the regulation of cellular iron, calcium and ROS homeostasis, lipid, and glucose homeostasis, as well as cancer cell proliferation and tumor growth [20]. Generally, the function of Fe-S clusters is the transfer of electrons. Recent investigations also suggest additional functions of different Fe-S clusters, such as iron or oxygen sensing, substrate binding/catalysis, and gene expression regulation. Different diseases are known to be caused by a dysfunction of the biogenesis pathway of Fe-S clusters. Among those are Friedreich’s ataxia, glutaredoxin-5-deficient sideroblastic anemia, ISCU myopathy, and *ABCB7* sideroblastic anemia/ataxia syndrome [19].

*CISD2* is primarily known to cause the neurodegenerative Wolfram syndrome 2 (WFS2) through a transcriptional splicing error, leading to hearing deficiency, blindness, diabetes mellitus, and a reduced life expectancy. Patients have been reported to develop bleeding intestinal ulcers and defective platelet aggregation without presenting with diabetes insipidus or psychiatric disorders, which would be clinical presentation of the related Wolfram syndrome 1 (WFS1) [59]. Cell models have shown that NAF-1 transfers its [2Fe-2S] clusters to an apo-receptor protein and transfers iron to intact mitochondria [60]. A study performed in mice showed that Cisd^(−/−)^ mice suffered from phenotypic premature aging, blindness, distortion of the skeleton, hemorrhages, and muscular and nerve degeneration. Furthermore, a damage to the mitochondria and an increase of autophagy was seen. These results show a close link to the WFS2 phenotype and suggest that mitochondrial dysfunction is causal for the disease [61].

The study, however, did not include blood testing, so at this point, a possible anemia of the Cisd^(−/−)^ mice cannot be excluded or confirmed. Further investigation is necessary to determine if iron deficiency and/or anemia occurs in these mice. In regard to the patient, he only suffers from a heterozygous mutation of *CISD2*, but WFS2 is caused by a homozygous mutation.

*KRI1* is located on chromosome 19. Little is known about the gene and its function to date. It has been investigated in a zebrafish model (*KRI1l*). The results suggest that it plays an important role in definitive hematopoiesis. A defective gene leads to dysregulated ribosome biogenesis, which is responsible for pathologies such as Diamond-Blackfan anemia and bone marrow failure in humans [26].

To conclude, we present a case of unknown, severely iron deficient anemia. Gene analysis led to the hypothesis of two potential causative genes with mutations. For sure, the definitive cause cannot be concluded at 100 % Further investigation of *CISD1* and *KRI1* in mice and in humans will have to unravel these findings.

In order to treat our patient, it is important to know the genetic mutation as well as the pathophysiology causing anemia. While probably not applicable to date, gene therapy is becoming an option for the treatment of anemia in the future [62]. Already, patients with inherited bone marrow failure syndromes are diagnosed by DNA sequencing, either WES or target sequencing, and are effectively treated [63].

## Figures and Tables

**Table 1:** Results of laboratory tests of the patient. The table shows the results of repeated laboratory tests in order to monitor the patient’s clinical state and his therapy and to exclude possible differential diagnoses of iron deficiency anemia. The variations in the measured parameters can be explained through the repeated substitution of RBC concentrates, as well as oral and intravenous iron. Testing of homeostasis confirmed no hemorrhagic diathesis. Molecular markers of acute myeloic anemia (AML) were negative. RBC: red blood count, Hct: hematocrit, MCV: mean corpuscular volume, MCH: mean corpusucular hemoglobin, MCHC: mean corpuscular hemoglobin concentration, Ret He: reticulocyte hemoglobin equivalent, HBDH: Hydroxybutyrat-dehydrogenase, WBC: white blood count, HGB: hemoglobin, PLT: platelets.

	06/12/ 10	07/07/ 11	20/06/ 12	20/12/ 12	25/04/ 13	29/08/ 13	19/12/ 13	08/05/ 14	05/03/ 15
RBC, (mcL) norm 4.44–5.61	3.15	4.8	3.48	3.91	3.98	4.33	4.45	3.58	4.32
Hemoglobin (g/dL): 13.5–16.9	8.6	13.0	9.4	9.8	11.2	11.2	10.9	9.0	11.2
(Hct, %): 40–49.4	27.7	39.1	28.8	31.1	33.7	36.0	34.9	29.4	35.2
MCV (fL): 81.8–95.5	87.9	81.5	82.8	79.5	84.7	83.1	78.4	82.1	81.5
MCH (pg): 27–32.3	27.3	27.1	27.0	25.1	28.1	25.9	24.5	25.1	25.9
MCHC (g/dL): 32.4–35	31.0	33.2	32.6	31.5	33.2	31.1	31.2	30.6	31.8
Reticulocyte count (%): 0.4–1.36	5.7	1.6	4.0	3.3	4.5	2.2		5.8	
Reticulocyte count (10^3^/mcL): 23–70	180.2	75.4	138	129	179	93		208	
Ret He (pg): 32,1–38.8	16.7	22.7	22.1	16.9	24.6	24.0		17.9	
Reticulocyte production index: 2–3	2.0		1.5	1.5	2.4	1.3		2.3	
Free hemoglobin (mg/dL): 0–15								6.7	
Ferritin (µg/L): 22–322	10	19	58	9	262	33	39	102	12
Serum iron (µg/dL): 80–150		44	23	21	346			29	34
Transferrin (mg/dL): 200–360			301	424	284				337
Transferrin saturation (%): 16–45			5	4	86				7
Haptoglobin (mg/dL)				153				120	
Erythropoetin (mU/mL): 6–23			174						
Coeruloplasmin (mg/dL): 20–60						21			
alpha HBDH (U/L): 72–182				180				190	
Zinc (mc/L): 750–1400					662				
Copper (mc/dL): 65–165					125				

**Table 2:** Additional laboratory testing. Due to the severity of the patient’s condition, additional tests were perfomed in order to diagnose him.

Date	Type of Examination	Results
19/07/2011 27/06/2012 17/05/2013	Molecular markers of AML examination with PCR	PMLRAR α (t(15;17)) amo negative PMLRAR α (t(15;17)) bmo negative AML1 ETO (t(8;21)) negative CBFB MYH11 (inv 16) negative BCRABL MAJOR p210 (t(9;22)) negative bcrabl minor p190 (t(9;22)) negative FLT3 ITD negative no NPM mutation detectable
08/05/2014	Hemostasis	vWF-antigen: 208,1% (norm: 45–200) Protein S activity: 212,8 % (norm: 60–120) PFA 100 EPI: > 250 s (norm: 85–165 s.) Factor V Leiden mutation: negative Prothrombin Mut. G20210A: negative Evaluation: no evidence for thrombophilic risk factors, no evidence for a Willebrand syndrome or a coagulopathy During Acetylsalicylic acid therapy sufficient prolongation of shutter speed, no evidence for aspirin resistance or thrombocytic hyperreactivity
06/12/2010	Blood typing	Blood group: 0 Rhesus factor: positive Rhesus system: CcD.ee Kell: negative Antibodies: negative Cytomegalovirus antibodies: IgG and IgM negative
08/05/2014	Blood typing	monospecific Coombs-test: IgG, IgA, IgM, C3d, C3c: negative AKS indirect Coombs Diamed: negative

**Table 3:** Follow up of laboratory results between 2019–2021.

Date	WBC (/mcL)	RBC (/mL)	HGB (g/dL)	HCT (%)	MCV (fL)	MCH (pg)	MCHC (g/dL)	PLT (mcL)	Ferritin (ng/mL)
23/1/19 *	5.5	4.5	12.7	38.1	84.7	28.2	33.3	188	167
30/1/19	4.5	4.51	12.7	38.3	84.9	28.2	33.2	186	
12/6/19	4.3	4.63	13.1	39.8	86.0	28.3	32.9	156	
26/6/19	4.3	3.78	10.6	33.0	87.3	28.0	32.1	163	551
3/7/19	4.6	4.81	13.6	41.3	85.9	28.3	32.9	162	
10/7/19	5.2	4.78	13.5	41.2	86.2	28.2	32.8	177	
20/11/19	5.9	4.96	14.2	42.2	85.1	28.6	33.6	174	418
4/12/19	5.2	4.83	13.8	41.6	86.1	28.6	33.2	144	
5/2/20	6.5	5.02	14.6	43.0	85.7	29.1	34.0	164	
22/2/20	5.5	4.88	14.0	42.2	86.5	28.7	33.2	164	605
4/3/20	5.4	5.15	14.9	44.3	86.0	28.9	33.6	156	
17/6/20	4.8	4.67	13.4	39.5	84.6	28.7	33.9	157	602
1/7/20	5.6	3.72	10.6	32.9	88.4	28.5	32.2	173	306
9/12/20	5.4	4.83	14.1	41.1	85.7	29.2	34.1	179	
16/3/21	9.2	2.70	7.8	25.1	93.0	28.9	31.1	166	573
9/6/21	5.7	4.95	13.7	41.8	84.4	27.7	32.8	159	
22/9/21	5.9	4.24	12.3	37.5	88.4	29.0	32.8	155	

In order to monitor the patient’s response to the treatment, laboratory testing is still performed on a regular basis. * Additional results: RDW: 17.4%, iron: 79 µg/dL, transferrin: 2.9 g/L, transferrin saturation: 19%.

**Table 4:** Examination results. Extensive diagnostic tests were performed in order to identify a cause for his iron deficiency. Repeated bone marrow examinations showed iron deficiency; the findings correlated with low grade myelodysplastic syndrome (MDS). A skin biopsy showed no signs of iron accumulation there. A scintigraphy was performed to exclude a gastrointestinal hemorrhage.

Date	Type of Examination	Results
27/07/2011	Bone marrow examination	Hematopoetic bone marrow with discrete dysnuclear stigmata of erythropoesis and of megakaryocytes as well as microfocal abnormal lymphoid infitration. Taking the clinical information into account, the findings can be correlated with MDS.
25/04/2013	Bone marrow examination	Myelogramm: Myeloblasts 0–2; promyelocytes 2–5, myelocytes 9–17, metamyelocytes 7–25, banded neutrophils 9–15, segmented neutrophils 4–11, eosinophils 1–5, basophils 0–1, monocytes 0–1, proerythroblasts 1–3, macroblasts 2–5, normoblasts 12–28, lymphocytes 7–22, plasmacells 0–4, reticulum cells 0–1, tissuebasophile mastcells 0–1, other blasts <5%. Granulopoesis: quantification normal, hypogranulation Megakaryopoesis: increased, hyposegmentation 5q Erythropoese: quant normal, dyserythropoesis Iron in bone marrow shows severe depletion. FACS analysis: no significant evidence for the presence of blasts (<5%) Diagnosis: Mild erythropoetic hyperplasia of the hematopoetic bone marrow with reticulocytic iron depletion, no infiltrations of a malign tumor, iron defeciency.
05/03/2015	Bone marrow examination	Maturing hematopoesis without significant signs of dysplasia, hemoglobin deficiency and significant deficiency of stored iron, no increase in blasts FACS analysis: no significant population of blasts, maturing hematopoesis
30/04/2013	Skin biopsy	Microscopy shows skin with unobtrusive epidermis and normal skin appendage, periadnexal lymphocytic infiltration. Diagnosis: mostly age-appropriate skin with no evidence of cutaneous accumulation.
08/05/2014	Sctintigraphy (Tc-99m-Ultra-Tag) with SPECT/CT for the detection of a gastrointestinal hemorrhage	No evidence for a gastrointestinal bleeding. The constant presentation of the intestinal loop in the upper left abdomen cannot exclude a angiodysplasia (differential diagnosis: regional hyperemic intestinal loop). Comparison with morphologic imaging is recommended.

**Table 5:** Autosomal-dominant inheritance with variable expression of the phenotype.

Chromosome	Location	Ref	Alt	Gene	Change of Amino Acid	CADD Score	rsID	gnomAD_ Exome
1	114269137	G	A	*PHTF1*	p.P131S	23.6	-	-
1	118584464	C	T	*SPAG17*	p.E1006K	21	rs200539422	0.00008064
2	179456326	C	G	*TTN*	p.V11009L	22.9	-	-
4	88731867	C	T	*IBSP*	p.T119M	25	rs866752943	0.00003591
4	103806433	C	T	*CISD2* SLC9B1 *	p.C462Y p.A55V	29.2	rs866399747	-
5	179751867	G	A	*GFPT2*	p.R209W	34	rs753649376	0.00002686
6	33382090	C	A	*PHF1*	p.R275S	23.7	-	-
8	144550669	C	T	*ZC3H3*	p.R663Q	23.1	rs754727062	-
19	6467566	C	A	*DENND1C*	p.Q741H	23.6	rs778032935	-
19	10670511	C	T	*KRI1*	p.R307H	32	rs1202991375	0.000008976
19	58967096	G	A	*ZNF324B*	p.C262Y	25.2	rs774176207	0.00005501

* Overlapping exons.

**Table 6:** De novo mutations.

Chromosome	location	Ref	Alt	Gene	Change of Amino Acid	CADD Score	rsID	gnomAD Exome
1	7792598	C	A	*CAMTA1*	p.A45E	22.5	rs367848023	0.000045
1	155240725	C	T	*CLK2*	p.R15Q	24.2	rs776199117	0.000008952

**Table 7:** Candidate genes and their genetic function. The table shows all candidate genes that were found by the WES analysis. Screening of the literature revealed that two of these genes seem to be the strongest considering their clinical relevance, CISD2 and KRI1.

Gene	Gene Name	Gene Function and Phenotypes of Genetic Mutations	Literature
*PHTF1*	Putative homeodomain transcription factor 1	Main expression in testes, associated with rheumatoid arthritis and type 1 diabetes, overexpression in acute lymphoblastic leukemia	[14] [15]
*SPAG17*	Sperm associated antigen 17	Organization of microtubuli and function of the axoneme; mutations cause primary ciliary dyskinesia, SNP cause skeletal malformations of the limbs in mice	[16]
*TTN*	Titin	Encodes protein of striated muscle, mutations cause neuromuscular diseases	[17]
*IBSP*	Integrin binding sialoprotein	Encodes a major structural protein of bone matrix, discussed as a factor in the development of osteoarthritis	[18]
*CISD2*	CDGSH iron sulfur domain 2	Encodes zinc finger protein NAF-1, a recently discovered member of the NEET protein family in the endoplasmatic reticulum, involved in iron and ROS homeostasis, as well as autophagy and apoptosis, cause of Wolfram syndrome 2	[19] [20]
*SLC9B1*	Solute carrier family 9 member B1	Encoded protein is a sodium/hydrogen exchanger and transmembrane protein which is primarily expressed in testes, essential for sperm motility and fertility	[21]
*GFPT2*	Glutamine-fructose-6-phosphate transaminase 2	Controls flux of glucose into the hexamine pathway, common variants are associated with type 2 diabetes and diabetic nephropathy	[22]
*PHF1*	PHD finger protein 1	Encodes polycomb group protein, functions in transcriptional repression of homeotic genes, recruited to double streak breaks, promotes cell proliferation, invasion and tumorigenesis, key factor for tumor progression	[23]
*ZC3H3*	Zinc finger CCCH domain-containing protein 3	Relevant for export of polyadenylated mRNAs from the nucleus, highly expressed in bone marrow	[24]
*DENND1C*	Differentially expressed in normal and neoplastic cells domain 1C	Guanine nucleotide exchange factor for the early endosomal small GTPase, which regulates endosomal membrane trafficking, involved in actin polymerization, potential role in glucose transport and homeostasis	[25]
*KRI1*	KRI1 homolog	DNA binding, importance in hematopoiesis	[26]
*ZNF324B*	Zinc finger protein 324B	Transcriptional regulation, discussed as a reference gene in human pluripotent stem cells	[27]
*CAMTA1*	Calmodulin binding transcription activator 1	Mainly expressed in adult brain tissue, encodes a transcription factor and is discussed as a tumor suppressor	[28]
*CLK2*	Cdc-like kinase 2	Phosphorylation of serine/threonine and tyrosine-containing substrates, importance in cell cycle and different malignancies, overexpression in breast cancer, modulates hepatic gluconeogenesis and fatty acid oxidation	[29]
*HCLS1*	Hematopoietic lineage cell-specific substrate 1	Antigen receptor signaling for clonal expansion and deletion in lymphoid cells, overexpression in B-chronic lymphocytic leukemia	[30]
*RP1L1*	Retinitis pigmentosa 1-like 1 protein	Differentiation of photoreceptor cells, discussed as cause for occult macular dystrophy	[31]

## Data Availability

As privacy issues are present, data is not shared publicly. Upon contact to the senior author, data availability can be discussed. Data sharing will only be performed in accordance with consent provided by participants on the use of confidential data.
